# The meiotic regulator JASON utilizes alternative translation initiation sites to produce differentially localized forms

**DOI:** 10.1093/jxb/erx222

**Published:** 2017-07-11

**Authors:** Simon Cabout, Megan P Leask, Shiny Varghese, Jun Yi, Benjamin Peters, Lei Liu Conze, Claudia Köhler, Lynette Brownfield

**Affiliations:** 1 Department of Biochemistry, University of Otago, Dunedin, New Zealand; 2 Department of Plant Biology, Uppsala BioCenter, Swedish University of Agricultural Sciences and Linnean Center for Plant Biology, Uppsala, Sweden

**Keywords:** Alternative translation initiation, Golgi, JASON, leaky ribosome scanning, meiosis, plasma membrane, subcellular localization, unreduced gametes

## Abstract

The JASON (JAS) protein plays an important role in maintaining an organelle band across the equator of male meiotic cells during the second division, with its loss leading to unreduced pollen in Arabidopsis. In roots cells, JAS localizes to the Golgi, tonoplast and plasma membrane. Here we explore the mechanism underlying the localization of JAS. Overall, our data show that leaky ribosom scanning and alternative translation initiation sites (TISs) likely leads to the formation of two forms of JAS: a long version with an N-terminal Golgi localization signal and a short version with a different N-terminal signal targeting the protein to the plasma membrane. The ratio of the long and short forms of JAS is developmentally regulated, with both being produced in roots but the short form being predominant and functional during meiosis. This regulation of TISs in meiocytes ensures that the short version of JAS is formed during meiosis to ensure separation of chromosome groups and the production of reduced pollen. We hypothesize that increased occurrence of unreduced pollen under stress conditions may be a consequence of altered usage of JAS TISs during stress.

## Teaser

Leaky ribosome scanning produces a long Golgi-localized and a short plasma membrane-localized version of the JAS protein, with the short version required during meiosis to ensure products are reduced.

## Introduction

The correct subcellular localization of a protein is an essential aspect of its function and is vital for cellular performance. Proteins are directed to different subcellular locations largely based on their primary amino acid (AA) sequence and secondary structure. Many proteins contain characterized targeting signals such as N-terminal transit peptides for mitochondrial and plastid import, N-terminal signal peptides for entry into the endoplasmic reticulum and the secretory pathway or internal nuclear localization signals (reviewed in Bauer *et al.*, 2015). Additionally, many proteins lack defined signals but are still targeted to specific subcellular compartments. For example, peripheral membrane proteins lack transmembrane domains and defined targeting signals yet are localized to specific membranes (Ghosh *et al.*, 2008).

Studies with fluorescent reporter proteins and organelle-specific proteome analyses indicate that many genes encode proteins that localize to multiple places within a cell (Small *et al.*, 1998; Silva-Filho, 2003; Lilley and Dupree, 2007; Carrie and Whelan, 2013). These proteins may contain ambiguous or multiple targeting signals. While multiple signals may act competitively on a single protein, they can also be found on different versions of a protein encoded by the same gene. Mechanisms to produce these different proteins include transcriptional regulation or alternative splicing producing multiple transcripts, translational control using alternative translation initiation sites (TISs) and protein cleavage (Silva-Filho, 2003).

We have previously shown that the JASON (JAS) protein in Arabidopsis is located in multiple membranes within cells, namely the Golgi, *trans*-Golgi network, tonoplast and plasma membrane (Brownfield *et al.*, 2015). The molecular function of the JAS protein is unknown, but cytoplasmic organization is disturbed in male meiotic cells lacking JAS (Brownfield *et al.*, 2015). This disturbance includes the loss of a band of organelles that usually persists between two sets of chromosomes that are in a common cell during the second meiotic division. As a consequence of this loss, the two meiotic spindles can move, commonly leading to poles of the two spindles being close, forming either tripolar or parallel spindles. Subsequently, chromosomes from each spindle are incorporated into a single cell. Such cells are termed unreduced, as the chromosome content has not been halved during meiosis. These unreduced meiotic products develop into functional pollen capable of fertilization (Erilova *et al.*, 2009; De Storme and Geelen, 2011). Such unreduced gametes are believed to have played an important role in ploidy increases during plant evolution and the creation of many modern crop varieties, and are utilized by plant breeders to overcome ploidy barriers.

Here we explore the mechanism controlling the localization of JAS to multiple locations. We reveal that there are two versions of JAS that localize to different subcellular compartments: a longer Golgi-localized form with the N-terminal extension and a shorter plasma membrane-localized form. We show that the N-terminal region of the longer form is both necessary and sufficient for Golgi localization, and that basic residues play a role in this localization. By altering the nucleotides immediately upstream of the first ATG the production of the Golgi-localized protein was favored, strongly suggesting that leaky ribosome scanning leads to the use of alternative TISs producing the two forms of JAS. The ratio of the two forms of JAS is likely to be developmentally regulated with the short, plasma membrane-localized form being predominant and functional in male meiotic cells.

## Materials and methods

### Plant material

*Arabidiopsis thaliana* plants in the Columbia background with mutant plants containing the *jas-3* allele (SAIL_813_H03; Erilova *et al.*, 2009) were used. Primers to verify the T-DNA insertions by PCR are shown in Supplementary Table S1 at *JXB* online. Arabidopsis and *Nicotiana benthamiana* plants were grown at 20–21 °C under a 16 h light–8 h dark cycle, with ~70% relative humidity. Transgenic Arabidopsis lines were generated using *Agrobacrerium tumefaciens* strain GV3101 and a modified floral drop method (Martinez-Trujillo *et al.*, 2004). Fluorescent markers were SYP32-mCherry (Golgi), VAMP711-mCherry (tonoplast) and PIP1;4-mCherry (plasma membrane) from the Wave line collection (Geldner *et al.*, 2009).

### Plasmid construction

**Fig. 1.**
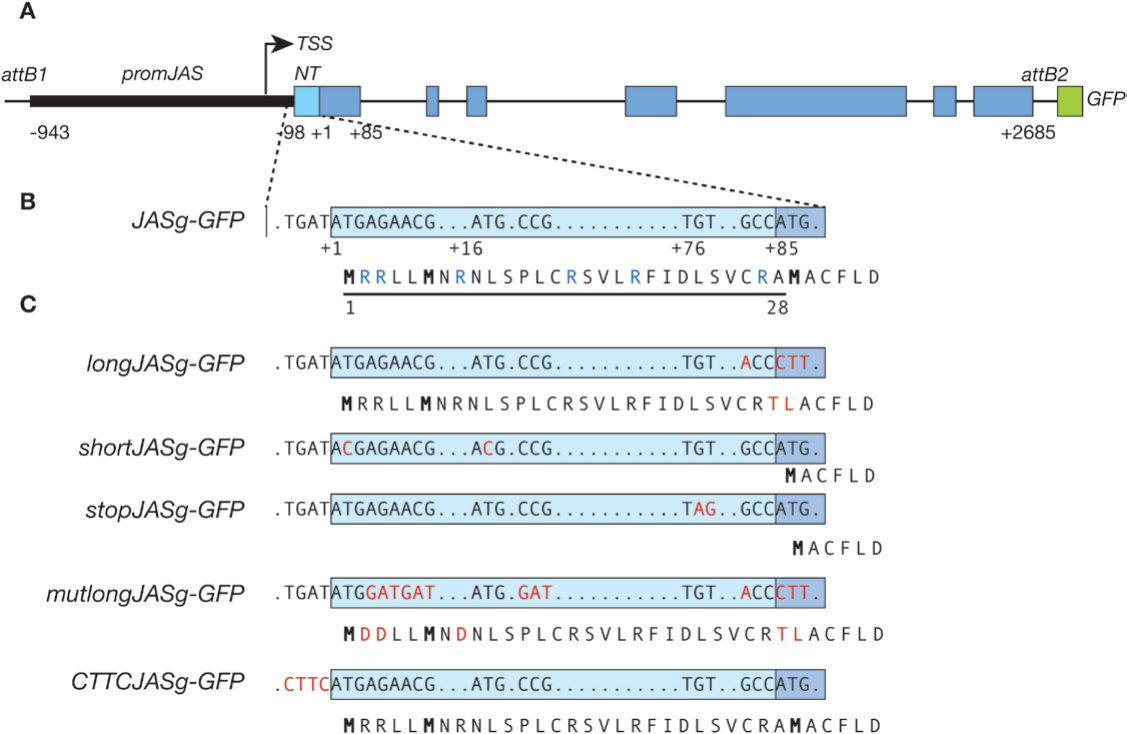
Schematic diagram of the constructs used to investigate JAS localization. (A) The *JASg–GFP* construct. The genomic region of *JAS* from 943 bp upstream of the predicted translation initiation ATG (+1;TAIR10) to immediately before the stop codon (2685 bp downstream of the ATG) was cloned upstream of *GFP* (green) between the *att* Gateway cloning sites of the *PMDC107* vector (Curtis and Grossniklaus, 2003). The promoter region is indicated by a thin black bar, exons by rectangular boxes and introns by thin black lines. The predicted transcription start site (TSS; TAIR10) is indicated by an arrow. The region encoding the N-terminus is shaded light blue with the remainder of the coding sequence in dark blue. (B) Enlargement of the region encoding the N-terminus. The position of ATGs is marked. The AA sequence of the encoded protein is indicated below with the 28 AA N-terminal region underlined, methionine residues bold and basic AAs blue. (C) Site-directed mutagenesis was used to create constructs encoding long or short version of JAS or to alter the sequence before the first ATG. Changes to the nucleotide sequence and the encoded peptide are highlighted in red.

A genomic fragment of *JAS* (*JASg*) from 943 bp upstream of the TIS (TAIR10) to immediately before the stop codon (+2685) was amplified from genomic DNA (primers in Supplementary Table S1) using Kapa HiFi polymerase (Kapa Biosystems), subcloned into *pENTR-D-TOPO* (Thermo Fisher Scientific) and verified by sequencing. The subsequent *pENTR-D-TOPO:JASg* was used as a template for site directed mutagenesis using the QuikChange II kit (Agilent Technologies) and primers in Supplementary Table S1 to generate the constructs shown in Fig. 1. The fragments were transferred to *PMDC107* (Curtis and Grossniklaus, 2003) in frame with *mGFP6* via an LR reaction with LR Clonase II (Thermo Fisher Scientific).

The *pUBQ14:longJASc-GFP*, *pUBQ14:shortJASc-GFP*, *pUB Q14:JAS-LIKE-GFP*, *pUBQ14:NT-JAS-LIKE-GFP*, *pUBQ14:NT-GFP*, *pUBQ14:longMtrJR1c-GFP*, and *pUBQ14:shortMtrJR1c-GFP* constructs were generated by Gateway multisite cloning. DNA was amplified from Arabidopsis genomic DNA (*pUBQ14*) or inflorescence cDNA from Arabidopsis (*JASc*, *JAS-LIKE*, *JASNT*) or Medicago (*Medicago truncatula*) R108 (*longMtrJR1c* and *shortMtrJR1c*) using Phusion high fidelity polyermase (Finnzymes) and primers with suitable attachment sites (Supplementary Table S1). To join the JAS N-terminal region and JAS-LIKE, fragments were amplified separately and joined in a stitching PCR. PCR fragments were cloned into suitable pDONR vectors via BP reactions and verified by sequencing. A multipart LR reaction using LR Clonase II Plus (Thermo Fisher Scientific) and destination vector *pK7m34GW* (Karimi *et al.*, 2005) was used to generate expression vectors.

For the luciferase assay the region from 32 bp upstream to 165 bp downstream of the TIS was amplified from the appropriate *TOPO:JASg* vector using Platinum Taq DNA polymerase (Thermo Scientific) and primers with appropriate restriction enzyme sites (Supplementary Table S1). PCR products were cloned via restriction digest and ligation into *pGreenII 0800-LUCm* (Simpson *et al.*, 2010) and verified by sequencing.

### Complementation analysis

Complementation of the *jas* phenotype was assessed by counting tetrads, triads and dyads as described in Brownfield *et al.* (2015) and by observation of mature pollen size, which is larger for unreduced pollen (De Storme and Geelen, 2011), using an Olympus IX71/IX51 inverted microscope and 4′,6-diamidino-2-phenylindole (DAPI) filters.

### Confocal microscopy and colocalization analysis

**Fig. 2.**
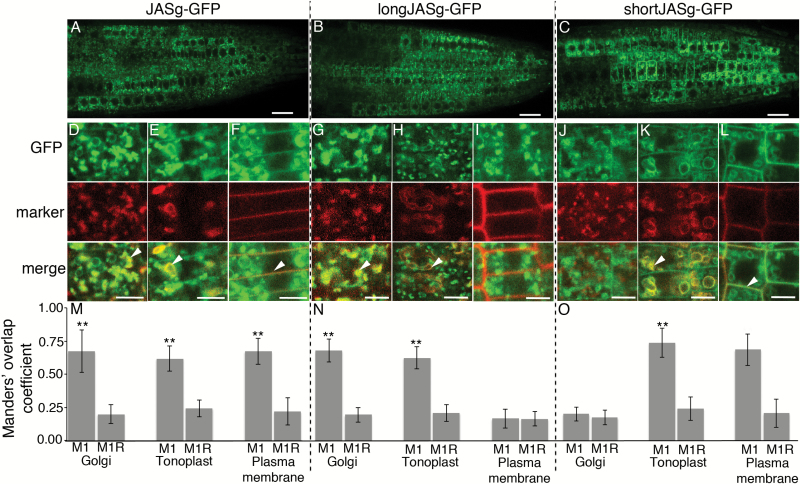
Subcellular localization of different forms of JAS–GFP. (A–C) CLSM images showing GFP fluorescence in whole root tips for JASg–GFP (A), longJASg–GFP (B) and shortJASg–GFP (C). (D–L) CLSM images showing colocalization of JASg–GFP (D–F), longJASg–GFP (G–I) and shortJASg–GFP (J–L) with fluorescent markers for the Golgi (D, G, J), tonoplast (E, H, K), and plasma membrane (F, I, L). The top panels show GFP fluorescence (green), the middle panels show marker fluorescence (red), and in the bottom panels colocalization can be visually assessed by the yellow color in merged images with examples highlighted with an arrowhead. Differences in marker morphology reflect differences in cell type and age due to the selection of cells being based on having both fluorescence signals that varied in location between roots. Scale bars: 10 μm (A–C); 5 μm (D–L). (M–O) Quantification of colocalization. For each combination of JAS–GFP and marker the mean (±SD) Manders’ overlap coefficient 1 value (M1) from 20 images is shown along with the mean (±SD) for the same red image with the green image randomized (M1R). **M1 value is significantly higher than the M1R value (*t*-test, *P*<0.001). Similar results were observed for two or three individual transgenic lines.

**Fig. 3.**
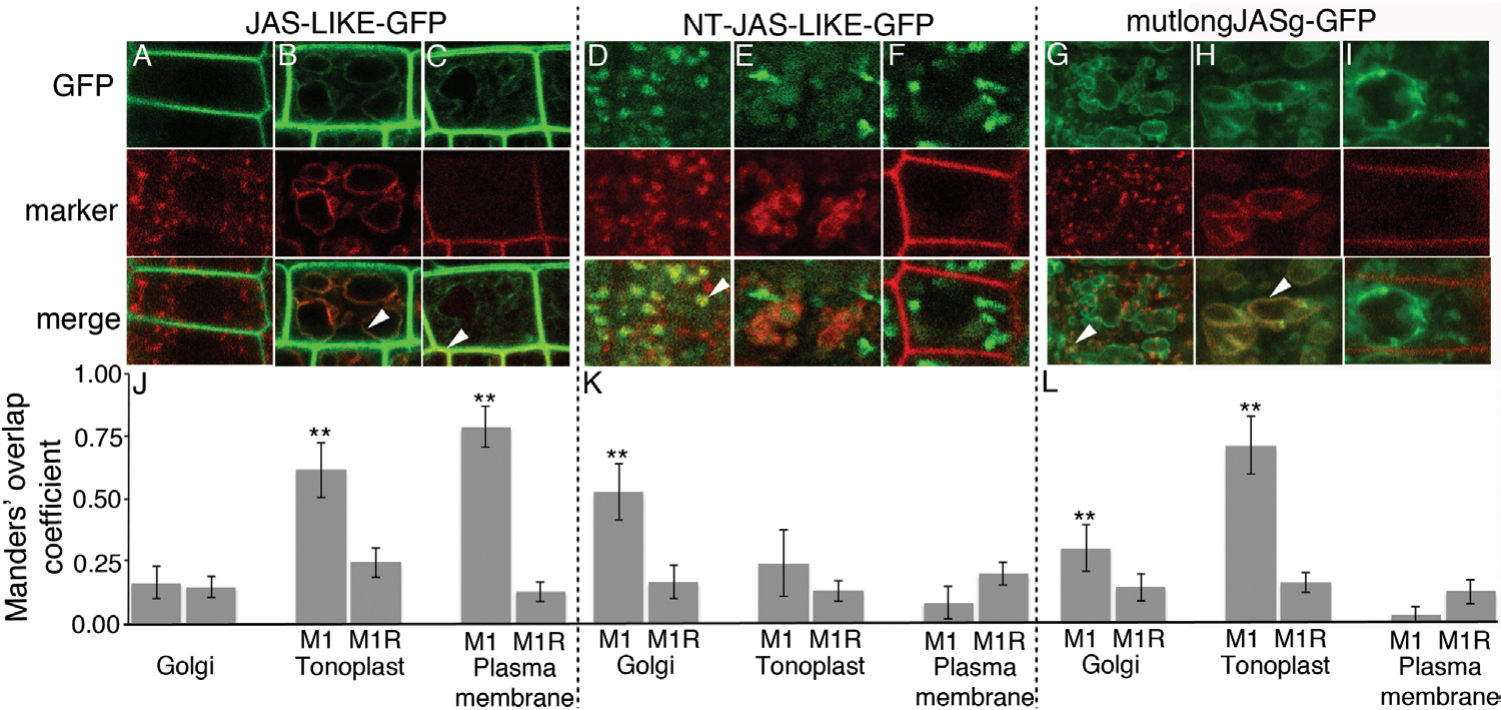
Subcellular localization of different versions of JAS-LIKE–GFP and JAS–GFP with a mutated N-terminal region. CLSM images showing colocalization of JAS-LIKE GFP (A–C), NT-JAS-LIKE–GFP (D–F) and mutlongJASg–GFP (G–I) with fluorescent markers for the Golgi (A, D, G), tonoplast (B, E, H), and plasma membrane (C, F, I) in root cells. The top panels show GFP fluorescence (green), the middle panels show marker fluorescence (red), and in the bottom panels colocalization can be visually assessed by a yellow color in merged images with examples highlighted with an arrowhead. Differences in marker morphology reflect differences in cell type and age due to the selection of cells being based on having both fluorescence signals that varied in location between roots. Scale bars: 5 μm. (J–L) Quantification of colocalization. For each combination of JAS–GFP and marker the mean (±SD) Manders’ overlap coefficient 1 value (M1) from 20 images is shown along with the mean (±SD) for the same red image with the green image randomized (M1R). **M1 value is significantly higher than the M1R value (*t*-test, *P*<0.001). Similar results were observed for two or three individual transgenic lines.

**Fig. 4.**
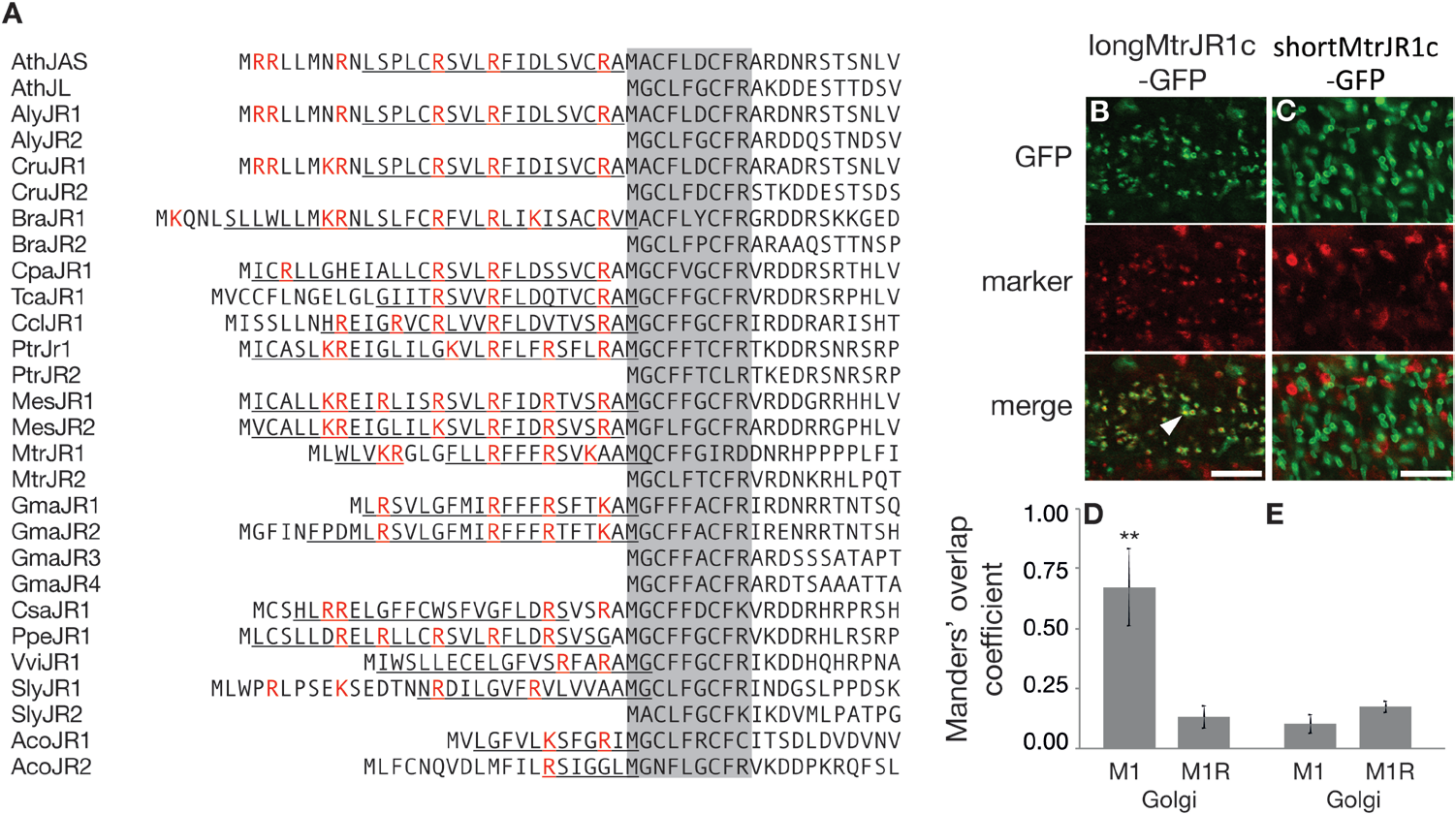
The N-terminal region of JAS-RELATED (JR) proteins in eudicots. (A) Alignment of the N-terminal region of JR proteins from throughout the eudicots. A conserved region following the M at position 29 in Arabidopsis JAS is shaded. All species have a least one long JR protein with an N-terminal extension. Basic residues in these N-terminal extensions are highlighted in red and predicted α-helices underlined. Species are as follows: Ath, *Arabidopsis thaliana*; Aly, *Arabidopsis lyrata*; Cru, *Capsella rubella*; Bra, *Brassica rapa*; Cpa, *Carica papaya*; Tca, *Theobroma cacao*; Ccl, *Citrus clementine*; Ptr, *Populus trichocarpa*; Mes, *Manihot esculenta*; Mtr, *Medicago truncatula*; Gma, *Glycine max*; Csa, *Cucumis sativus*; Ppe, *Prunus persica*; Vvi, *Vitis vinifera*; Sly, *Solanum lycopersicum*; Aco, *Aquilegia coerulea*. Gene identifiers are in Supplementary Table S2. (B, C) CLSM images of Arabidopsis root cells showing colocalization of longMtrJR1c–GFP (B) and shortMtrJR1c–GFP (C) with a fluorescent marker for the Golgi. The top panels show GFP fluorescence (green), the middle panels show Golgi marker fluorescence (red), and in the bottom panels colocalization can be visually assessed by the yellow color in merged images with an example highlighted with an arrowhead. Scale bars: 5 μm. (D, E) Quantification of colocalization. For each combination of GFP and Golgi marker the mean (±SD) Manders’ overlap coefficient 1 (M1) value from 15 images is shown along with the mean (±SD) for the same red image with the green image randomized (M1R). **M1 value is significantly higher than the M1R value (*t*-test, *P*<0.001). Similar results were observed for two individual transgenic lines.

**Fig. 5.**
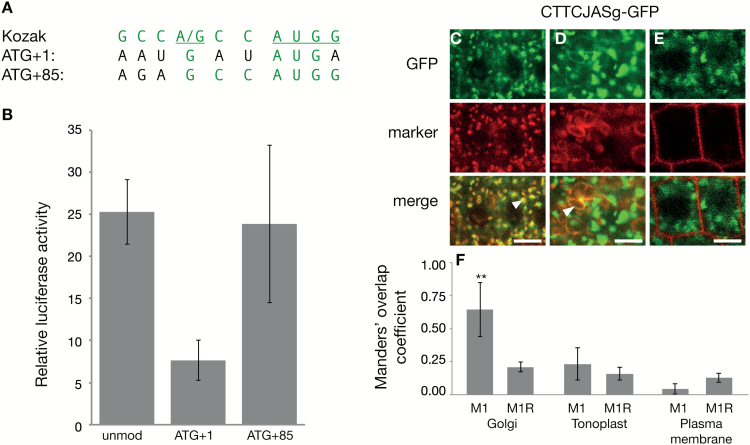
Leaky ribosome scanning leads to the multiple forms of JAS. (A) The nucleotides surrounding the AUG at position +1 and +85 of the *JAS* mRNA. The Kozak sequence for strong ribosome recognition is shown on top in green with the most important nucleotides underlined. The sequence surrounding the AUGs at +1 (AUG+1) and +85 (AUG+85) is shown below with nucleotides identical to the Kozak sequence highlighted in green. (B) Transient luciferase assay using the *CaMV35S* promoter and the last 32 bp of the *JAS* 5′-UTR driving expression of the 5′-end of *JAS* fused to the luciferase gene lacking an ATG. Luciferase activity was measured 3–4 d after infiltration into *N. benthamiana* leaves and values were normalized to luciferase without an ATG. Using the unmodified (unmod) version of the 5′-end of *JAS*, translation could start from the ATG at position +1 or +85. Translation initiation was limited to the ATG at position +1 (ATG+1) or position +85 (ATG+85) by mutating other ATGs. Bars show the mean (±SE) of three biological replicates each with three leaves. (C–E) Images showing colocalization of CTTCJASg–GFP with fluorescent markers for the Golgi (C), tonoplast (D), and plasma membrane (E). The top panels show GFP fluorescence (green), the middle panels show marker fluorescence (red), and in the bottom panels colocalization can be visually assessed by a yellow color in merged images with examples highlighted with an arrowhead. Scale bars: 5 μm. (F) Quantification of colocalization. For each combination of GFP and marker the mean (±SD) Manders’ overlap coefficient 1 value (M1) value from 20 images is shown along with the mean (±SD) for the same red image with the green image randomized (M1R). **M1 value is significantly higher than the M1R value (*t*-test, *P*<0.001). Similar results were observed for two individual transgenic lines.

For observing JAS–GFP in roots, seedlings were grown for 5–6 days on half-strength Murashige and Skoog agar before root tips were excised and mounted in water. For plasmolysis, roots were incubated for 1 h in 0.8 M mannitol then stained with 10 μM propridium iodide. Roots were viewed with an Olympus FluoView 1000 confocal microscope, using eGFP and Alexafluor 594 channels (Figs 2–5) or a Zeiss 780 confocal laser scanning microscope (Supplementary Fig. S3). Dwell time varied depending on signal strength and images are the average of four scans (Kalman 4). Colocalization was assessed using Manders’ overlap coefficient 1 (M1; Manders *et al.*, 1993), which determines the proportion of pixels with a signal in the red channel (marker) that also contain a signal in the green channel (JAS–GFP), using the JACoP plugin through ImageJ (Bolte and Cordelières, 2006). Manders’ overlap coefficient 2 and Pearson’s coefficient were not used due to the multiple locations of JAS–GFP (Brownfield *et al.*, 2015). As the intensity of JAS–GFP and the markers varied greatly between cells within a root, we analysed three to six cells where both markers had relatively strong and even expression. This meant different regions of roots were analysed for different combinations of JAS–GFP fusion proteins and markers, and consequently the morphology of the organelles varied. Additionally, for the plasma membrane marker, only images where the plasma membrane was separated from the internal organelles were selected. For each channel noise was removed using a median filter set at 2.0 pixels and the threshold level set automatically in the JACoP plugin, or manually adjusted when there was high background. Once Manders’ overlap coefficient was calculated, the green channel was randomized using Costes’ randomization in the JACoP plugin and Manders’ overlap coefficient was calculated again for the red and randomized green images giving M1R. This was repeated for up to 20 images for each construct and marker.

Confocal imaging of meiocytes and roots containing JASc–GFP constructs was as described in Brownfield *et al.* (2015).

### Luciferase assay

Vectors were transformed by electroporation into *A. tumefaciens* strain GV3101 containing the pSOUP-P19 helper plasmid (Hellens *et al.*, 2005). Transformed strains grown overnight on LB medium were suspended in infiltration medium (1 mM MgCl_2_ and 10 μM acetosyringone) to an OD_600_ of 0.5. After 2 h at room temperature, *A. tumefaciens* cells were infiltrated into *N. benthamina* leaves. Leaf disks of 7 mm were harvested 3–4 d after infiltration and homogenized in 50 μl 1× Cell Lysis Buffer (Targeting Systems). *Renilla* luciferase activity was measured sequentially using a CLARIOStar® microplate reader with injection of 50 μl Luciferase Assay Reagent (Promega) followed by 50 μl Renilla Luciferase Stop and Glo® reagent (Promega). Assays were conducted on three separate occasions to give three biological replicates with three leaves sampled on each occasion.

### Bioinformatics

Predictions of the subcellular localization of the JAS protein utilized TargetP (Emanuelsson *et al.*, 2000), TMpred (Hofmann and Stoffel, 1993), TMHMM (Krogh *et al.*, 2001), HMMtop (Tusnády and Simon, 1998), and SLP-Local (Matsuda *et al.*, 2005). Secondary structure prediction for JAS and JAS-RELATED (JR) proteins was conducted using PSIPRED (Jones, 1999).

JR proteins were identified by TBLASTN searches of angiosperm genomes in Phytozome (Goodstein *et al.*, 2012) and visually confirmed to contain the C-terminal plant conserved domain (Erilova *et al.*, 2009). N-terminal regions were manually aligned.

### Ribosome protection data analysis

Ribosome protection data from Juntawong *et al.* (2014) were downloaded from the Gene Expression Omnibus (GEO) database (www.ncbi.nlm.nih.gov/geo) under accession no. GSE50597. The raw reads were quality and adapter trimmed using cutadapt (Martin, 2011). The trimmed reads were mapped to the Arabidopsis genome sequence (TAIR10) using Bowtie2 (Langmead and Salzberg, 2012) with default settings. After merging the aligned reads from replicates, the histogram of normalized coverage (reads per million, RPM) of *JAS* in different samples were visualized using the R package Gviz from Bioconductor (Hahne and Ivanek, 2016).

## Results

### Different forms of JAS localize to the Golgi and plasma membrane in Arabidopsis roots

To investigate the mechanism of JAS localization to the Golgi, tonoplast and plasma membrane (Brownfield *et al.*, 2015), we first looked for known targeting signals in the JAS AA sequence, but found no known targeting signals for these subcellular locations. We then looked at the predicted secondary structure of JAS (Supplementary Fig. S1). The N-terminal region of JAS is rich in basic AA residues and is predicted to form an α-helix, while the remainder of the JAS protein was predicted to be unstructured, although with some α-helices. We therefore decided to focus on the N-terminus.

To elucidate the role of the N-terminal region in JAS localization, we cloned the *JAS* genomic region including the promoter and coding region upstream of the green fluorescent protein (GFP) sequence (*JASg-GFP*; Fig. 1A, B). We then generated forms of JAS with and without the N-terminal peptide by site directed mutagenesis. We altered an ATG and surrounding context at position +85 of *JASg-GFP*, allowing translation only from the upstream ATGs and thus formation of a long JAS protein (*longJASg-GFP*; Fig. 1C). We also generated a short version of *JAS-GFP* without the N-terminal peptide by mutating the ATGs at positions +1 and +16, enforcing translation to begin at the ATG at position +85 (*shortJASg-GFP*; Fig. 1C). A second short version was generated by introducing a stop codon at position +76 (*stopJASg-GFP*; Fig. 1C).

Fluorescence was analysed by confocal laser scanning microscopy (CLSM) in 5- to 6-day-old roots of transgenic Arabidopsis seedlings. For each fusion protein, GFP was detected in cells behind the root tip, although with varying fluorescence intensity in different cells and cell files in individual roots (Fig. 2A–C and Supplementary Fig. S2A). This similar expression pattern shows that altering the ATGs did not influence *JAS* promoter activity.

To determine the subcellular location of the different JAS–GFP proteins, transgenic plants were crossed with markers for the Golgi (SYP32–mCherry), tonoplast (VAMP711–mCherry) and plasma membrane (PIP1;4–mCherry) (Geldner *et al.*, 2009). Colocalization was quantified using Manders’ overlap coefficient 1 (M1) and compared with a coefficient calculated using a randomized green signal (M1 random; M1R). Consistent with previous data (Brownfield *et al.*, 2015), the unmodified genomic version JASg–GFP visually partially colocalized with all three markers (Fig. 2D–F), and each M1 value was significantly higher than M1R (Fig. 2M), indicating colocalization. The longJASg–GFP fusion showed an altered subcellular localization pattern, partially colocalizing with the Golgi and tonoplast markers but not with the plasma membrane marker (Fig. 2G–I, N). In contrast, shortJASg–GFP did not colocalize with the Golgi marker, but instead overlapped with the tonoplast and plasma membrane markers (Fig. 2J–L, O). Similar results were observed for stopJASg–GFP (Supplementary Fig. S2B).

We also generated transgenic lines expressing GFP fused to a version of the *JAS* cDNA with the N-terminal region (beginning at the ATG at position +1; *longJASc-GFP*) and a truncated version without the N-terminal sequence (beginning at the ATG at position +85; *shortJASc-GFP*) under control of the *UBQ14* promoter. When JAS contained the N-terminal sequence, a similar pattern to the longJASg–GFP was observed, with JAS being localized in organelles that overlap with the Golgi marker and also appearing to be in the tonoplast, but not in the plasma membrane (Supplementary Fig. S3A). The shortJASc–GFP showed a localization pattern similar to shortJASg–GFP, being visually present in the tonoplast and the plasma membrane, but not colocalizing with the Golgi marker (Supplementary Fig. S3B).

The localization pattern of the unmodified JAS (JASg–GFP) likely reflects that of the endogenous JAS protein, as it is encoded by a long genomic fragment of *JAS* with the C-terminal GFP being distant from the N-terminal region. As only the long version of JAS–GFP localizes to the Golgi and only the short version localizes to the plasma membrane, but JASg–GFP localizes to both, we conclude that long and short forms of JAS are normally produced in Arabidopsis roots. The JAS protein is also likely present at the tonoplast as a mixture of the long and short forms.

### The N-terminus of long JAS is sufficient for Golgi localization

The colocalization experiments using longJASg–GFP and shortJASg–GFP revealed that the N-terminal 28 AA of the long version is necessary for Golgi localization. We therefore addressed whether the N-terminus is also sufficient for Golgi localization.

The JAS-LIKE protein in Arabidopsis has a high degree of sequence similarity to JAS but lacks the N-terminal region of the long protein (Erilova *et al.*, 2009). We generated transgenic Arabidopsis plants expressing JAS-LIKE–GFP under the control of the *UBQ14* promoter. A strong GFP signal largely colocalized with the plasma membrane marker and a much weaker signal colocalized with the tonoplast marker in Arabidopsis root cells (Fig. 3B, C, J). Using plasmolysed cells we confirmed that the GFP signal colocalized with the plasma membrane and not the cell wall (Supplementary Fig. S4). There was no JAS-LIKE–GFP detected in the Golgi (Fig. 3A, J). These results indicate that the majority of JAS-LIKE is localized to the plasma membrane, so JAS-LIKE is a suitable protein to test the role of the JAS N-terminal peptide.

We then fused the N-terminal 28 AA of longJAS upstream of JAS-LIKE, creating NT-JAS-LIKE–GFP. Strikingly, the NT-JAS-LIKE–GFP protein was not localized to the plasma membrane or the tonoplast, but partially colocalized with the Golgi marker, although not all Golgi contained a strong GFP signal (Fig. 3D–F, K). Thus, the N-terminal region of JAS altered the localization of JAS-LIKE from the plasma membrane to the Golgi. Additionally, we also fused the N-terminal region of JAS onto GFP, leading to GFP being localized to the Golgi (Supplementary Fig. S5). The N-terminal 28 AA from JAS is therefore both necessary and sufficient for Golgi localization.

### The basic nature of the longJAS N-terminus is important for Golgi localization

The N-terminal region of the JAS protein is rich in basic AA residues (Fig. 1). We tested the importance of these basic residues by altering three arginine residues (R) to glutamic acid (D) in the longJASg–GFP construct, resulting in mutlongJASg–GFP (Fig. 1). The altered protein was predominantly localized to the tonoplast (Fig. 3G–I, L) with localization to the Golgi reduced compared with the longJASg–GFP protein with a significant difference in M1 values for longJASg–GFP and mutlongJASg–GFP (*t*-test *P*=5 × 10^−7^) (compare Fig. 2N and Fig. 3L). This suggests the alteration of basic to acidic residue at the N-terminus reduced localization to the Golgi. The change from basic to acidic residues in the N-terminus did not lead to plasma membrane localization (Fig. 3I, L). We thus conclude that the basic nature of the N-terminal region contributes to its ability to target proteins to the Golgi.

### An N-terminal Golgi-localization signal is functionally conserved in long eudicot JAS-RELATED proteins

We next asked if the N-terminal Golgi-localization signal is conserved in JAS-RELATED (JR) proteins. We focused on eudicots, as most eudicots undergo simultaneous cytokinesis during meiosis whereas monocots undergo successive cytokinesis and so do not require the organelle band in which JAS functions. We identified putative JAS homologs based on the C-terminal plant-conserved domain (Erilova *et al.*, 2009) from sequences available in Phytozome (Goodstein *et al.*, 2012). There was notable sequence conservation of the region of all these JR proteins following the ATG at the start of the shortJAS (position 29 in Arabidopsis longJAS) (shaded Fig. 4A). The JR proteins varied in whether they had an N-terminal extension beyond this conserved region. However, in all eudicot species examined there was at least one long JR protein with some species having a single long JR protein, others with one long and one short JR protein (like Arabidopsis) and some with two long JR proteins (Fig. 4).

While each eudicot has at least one long JR protein, there is not a high degree of sequence identity in the N-terminal extension (Fig. 4A). However, the sequences are commonly rich in basic AAs, the position of these is often similar, and each N-terminal extension is predicted to form an α-helix (Fig. 4A). Thus, the N-terminal extensions on JR proteins have some of the characteristics of the N-terminal Golgi-localization signal from the JAS protein from Arabidopsis.

To explore if the N-terminal region of JR proteins could act as a Golgi-localization signal, we decided to test the JR1 protein from the legume Medicago (*Medicago truncatula*). The N-terminal sequence of the Medicago JR1 (MtrJR1) protein differs from JAS but has the general characteristics described above (Fig. 4A). To follow the localization of MtrJR1, GFP fusion proteins using the cDNA beginning with the first ATG (*longMtrJR1c-GFP*) or second ATG (*shortMtrJR1c-GFP*) driven by the *UQB14* promoter were constructed. In Arabidopsis roots longMtrJR1c–GFP was partially localized to the Golgi (Fig. 4B, D), similar to the long version of the Arabidopsis protein (Fig. 2G, N). When the N-terminal extension was not present, the protein localization was altered with shortMtrJR1c–GFP localized in tubular structures throughout the cell (Fig. 4C). The identity of these structures is unknown, but they are unlikely to be Golgi as they had a different morphology and did not colocalize with the Golgi marker (Fig. 4C, E). Therefore, the N-terminal extension of MtrJR1 is necessary for Golgi-localization, suggesting that although there is sequence variation in the N-terminal extensions of JR proteins, they are functionally conserved to target the protein to the Golgi. However, unlike JAS in Arabidopsis, the short version of MtrJR1 was not localized to the plasma membrane (Fig. 4C), indicating that other localization signals may differ between the two proteins.

### Alternative translation initiation leads to the two versions of JAS

The presence of Golgi- and plasma membrane-localized proteins from the *JASg-GFP* construct indicates that one genomic region is giving rise to long and short versions of the JAS protein. As the long cDNA version of *JAS* (*longJASc-GFP*) encodes the same protein as the unmodified genomic form (*JASg-GFP*) yet they display different localization patterns (compare Fig. 2A, D–F with Supplementary Fig. S3A), we concluded that the two forms are unlikely to be generated by proteolytic cleavage of the protein. One of the major differences between the *JASg-GFP* and *longJASc-GFP* constructs is the 5′-UTR, with *JASg-GFP* containing the native *JAS* 5′-UTR and *longJASc-GFP* containing the 5′-UTR from the *UBQ14* promoter along with a 25 bp *attB1* Gateway cloning site immediately upstream of the ATG at +1. Due to these different sequence contexts around the ATG at +1 we hypothesized that leaky ribosome scanning could be responsible for the creation of the two forms of JAS. Leaky ribosome scanning occurs when the first ATG is sporadically skipped, often due to a suboptimal Kozak sequence context (Kozak, 1987), leading to the ribosome continuing to scan and initiating translation at a downstream TIS (often in a more optimal Kozak context) (Silva-Filho, 2003). Supporting this hypothesis, the nucleotides surrounding the first ATG of *JAS* are a suboptimal Kozak sequence for ribosome recognition while the ATG at position +85 is in a more optimal context (Fig. 5A).

In order for leaky scanning to arise, two AUGs in the mRNA must be able to act as TISs. To test if the AUGs at both +1 and +85 in *JAS* initiate translation, we used a transient luciferase assay where the end of the 5′-UTR and beginning of the coding region of JAS was cloned upstream of a luciferase gene lacking an ATG. When the vector containing an unmodified version of the *JAS* 5′-end was transiently expressed following infiltration of *N. benthamiana* leaves, there was an ~25-fold increase in luciferase compared with the vector with luciferase lacking an ATG (unmod, Fig. 5B). Therefore, the luciferase mRNA was being translated using the ATGs in *JAS*. When the 5′-end of *JAS* was modified so translation could only begin at an early ATG (ATG+1) or the later ATG (ATG+85), luciferase activity was again greater than the vector with luciferase lacking an ATG (Fig. 5B), demonstrating that both ATGs can act as TISs, consistent with leaky scanning. The level of luciferase translation from the ATG at +1 appeared lower than the level from the unmodified and ATG+85 vectors suggesting translation is not as efficient at this ATG, consistent with the weaker Kozak context; however, the difference was not significant (*t*-test).

Additionally, published ribosome protection data from Arabidopsis seedlings (Juntawong *et al.*, 2014) were analysed (Supplementary Fig. S6). There were small peaks of ribosome protected RNA close to the ATGs at both +1 and +85 of *JAS*, consistent with both being translation initiation sites and the long and short forms of JAS being translated in seedlings (Brar and Weissman, 2015). Interestingly, under different conditions there appeared to be differential use of the sites, with the ATG at +85 favored under normal levels of oxygen and an earlier site favored under hypoxia (Supplementary Fig. S6). However, due to low expression levels of *JAS* in whole seedlings, this difference cannot be reliably quantified.

To test if the context of the first ATG was sufficient to explain the differences in the localization pattern between JASg–GFP and longJASc–GFP, we used site-directed mutagenesis to alter the 4 bps upstream of the ATG in *JASg-GFP* to the same bases found in the *UBQ14-longJAScDNA-GFP* construct, resulting in *CTTCJASg-GFP* (Fig. 1). This modified construct provided a bright punctate expression pattern that colocalized with the Golgi marker (Fig. 5C). Fluorescence in the tonoplast was reduced for CTTCJASg–GFP compared with JASg–GFP, with a weak signal only detected in some cells (compare Fig. 2E, M with Fig. 5D, F). There was no fluorescence detected in the plasma membrane, or colocalization with the plasma membrane marker (Fig. 5E, F). These data reveal that changing the context of the first ATG in JAS leads to increased production of the Golgi-localized long form of JAS, and reduced production of the amount of the plasma membrane-localized protein, suggesting the long form was the predominant protein present. This is consistent with the two forms of JAS being generated through leaky ribosome scanning and alternative TISs with the change to CTTC providing high translation rates from the first ATG with a corresponding decrease in the use of the ATG at +85.

### The short version of JAS functions during meiosis

The above data suggest that there are two different forms of JAS present in plants cells, a longer version containing a basic N-terminus that is necessary and sufficient for Golgi localization, and a shorter version that is located in the plasma membrane. We next asked if the two different versions had different functional roles.

As the *jas* mutant does not have a root phenotype, we focused on meiotic cells where the *jas* mutation leads to formation of unreduced pollen (Erilova *et al.*, 2009; De Storme and Geelen, 2011; Brownfield *et al.*, 2015). As with our previous construct (Brownfield *et al.*, 2015), JASg–GFP complemented the *jas* meiotic phenotype in most transgenic lines (Table 1). Additionally, the shorter JAS version, expressed as both the genomic short version (*shortJASg-GFP* and *stopJASg*-*GFP*) and the short cDNA (*shortJASc-GFP*) complemented in about 50% of independent T1 lines (Table 1), showing that the plasma membrane-localized, short version is sufficient for JAS function in meiotic cells. However, the longJASg–GFP protein did not complement in 30 independent transgenic lines and the *longJASc*-*GFP* only rarely complemented (Table 1).

**Table 1.** Complementation of the *jas* phenotype by *JAS-GFP* constructs

Construct	Phenotype of plants ^*a*^			Percentage of lines complemented
	WT ^*b*^	*jas* ^*c*^	Total	
*JASg-GFP*	39	4	43	91
*shortJASg-GFP*	11	13	24	46
*stopJASg-GFP*	8	10	18	44
*shortJASc-GFP*	18	15	33	55
*longJASg-GFP*	0	30	30	0
*longJASc-GFP*	3	30	33	9

^*a*^The genotype of plants was *jas−/−* and confirmed by PCR.

^*b*^Wild type phenotype as assessed by the meiotic products being over 95% tetrads and/or pollen size being even.

^*c*^ *jas* phenotype as assessed by the meiotic products being less than 90% tetrads and/or pollen size being uneven with large pollen grains.

**Fig. 6.**
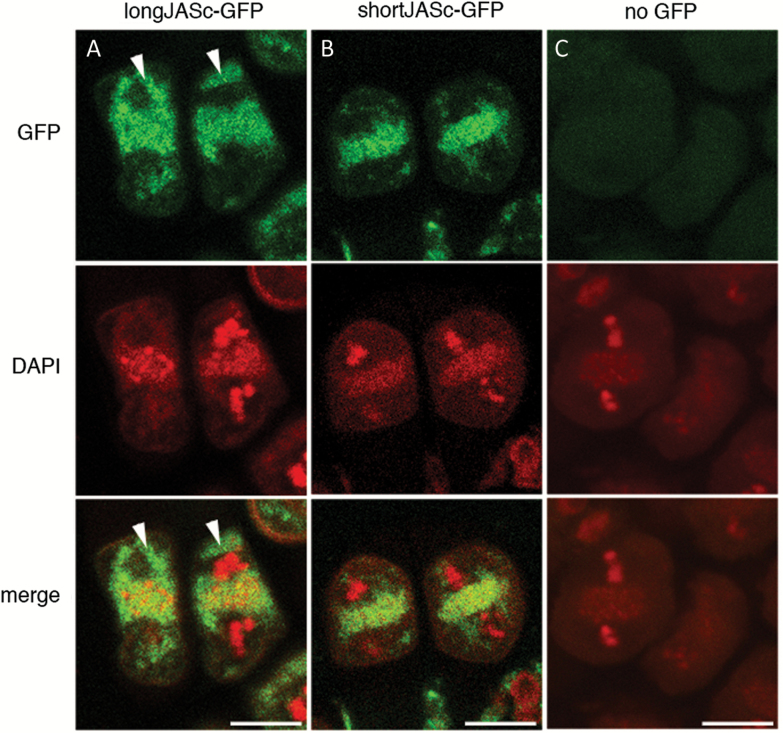
Location of long and short versions of JAS in meiotic cells. (A-C) CLSM images of longJASc–GFP (A), shortJASc–GFP (B) and control meiocytes without GFP (C) during the second meiotic division in Arabidopsis male meiocytes. Top panel shows GFP fluorescence (green), the middle panel shows DAPI staining of the chromosomes and organelle band and the bottom panel shows the merged images. Arrowheads show where longJASc–GFP is not overlapping with the organelle band. Scale bars: 10 μm.

We then investigated the location of the long and short version of JASc–GFP in male meiotic cells during meiosis II, where the *jas* mutant displays phenotypic differences from wild type (Brownfield *et al.*, 2015). LongJASc–GFP was located both in the organelle band that forms across the equator of cells at the end of the first meiotic division and in other regions of the cell, although it was excluded from the spindle region (Fig. 6A). In contrast, shortJASc–GFP was restricted to the organelle band and was not detected elsewhere in the cell (Fig. 6B).

## Discussion

Proteins localizing to more than one subcellular domain is a common occurrence in plants based on analyses of fluorescent fusion proteins and proteome studies on isolated organelles (Lilley and Dupree, 2007; Carrie and Whelan 2013). However, less is known about the details of how such proteins localize to specific places in the cell and the functional importance of the multiple forms (Carrie and Whelan 2013). The JAS protein is an example of such a protein with a fluorescent fusion protein localizing to the Golgi, tonoplast and plasma membrane in Arabidopsis root cells (Brownfield *et al.*, 2015). Here, we have investigated the mechanism behind the localization of the JAS protein to multiple subcellular locations in Arabidopsis and ask how this relates to its meiotic function.

### The JAS protein contains multiple localization signals

The JAS protein localizes to different cellular membranes. The most well studied mechanisms to localize proteins to membranes involve transmembrane domains with organelle-specific characteristics coupled with targeting information contained within cytosolic domains that interact with components of the vesicular trafficking machinery (Cosson *et al.*, 2013). However, the JAS protein does not contain any predicted transmembrane domains, or any characterized signals for targeting within the plant membrane system. The JAS protein is therefore likely to be a peripheral membrane protein recruited to membranes through interactions with membrane resident proteins or through post-translational modifications.

Here we have shown localization of JAS depends of the version of the protein made from a single genomic region, with a long protein localizing to the Golgi and a short version to the plasma membrane (Fig. 2). The difference between the long and short versions is an N-terminal extension of 28 AA on the long version that is both necessary (Fig. 2) and sufficient (Fig. 3) for Golgi localization, and is thus a Golgi localization signal. There are long JR proteins throughout the eudicots with an N-terminal extension, and this region of the Medicago JR1 protein also directs Golgi localization in Arabidopsis. While the N-terminal sequences vary in long JR proteins, they are rich in basic AAs and are predicted to form an α-helix. Thus, the Golgi localization signal of this region appears to lie in its general attributes rather than specific sequences. Consistent with the idea that basic AA residues are important, changing them to acidic ones reduced the ability of the N-terminal region to localize JAS to the Golgi (Fig. 3).

It is unknown how the N-terminal 28 AA extension acts in Golgi-localization, but as JAS is likely to be a peripheral membrane protein this region may be involved in protein–protein interactions with a Golgi-resident protein and/or contain a site for post-translational addition of lipids such as acetylation, palmitoylation, prenylation, or myristoylation giving the protein affinity for the lipid environment of the Golgi (Banfield, 2011). Similar localization mechanisms are used for peripheral membrane proteins such as the GTPase Arf1 involved in endomembrane trafficking (reviewed in Kahn, 2009). Arf1 is recruited from the cytoplasm upon GTP binding through the insertion of a myristoylated N-terminal α-helix into Golgi membranes.

The short version of JAS localizes to the plasma membrane. The absence of plasma membrane fluorescence with the longJAS–GFP protein indicates that the presence of the N-terminal region prevents plasma membrane localization. This could be due to the N-terminal region masking a second localization signal. Such masking of localization signals has been observed for other proteins, commonly the masking of nuclear localization signals leading to cytoplasmic retention of transcriptional regulators until required in the nucleus (Kaffman and O’Shea, 1999; Cyert, 2001). The conserved AA sequence at the N-terminus of plasma membrane localized shortJAS and JAS-LIKE proteins as well as JR proteins is similar to MGCXXS/T/C, a target site for myristoylation and palmitylation in other proteins (Aicart-Ramos *et al.*, 2011). In these proteins a myristate is covalently attached to the glycine residue when located at the N-terminus following the cleavage of the N-terminal Met residue and the adjacent cysteine residue is palmitoylated (Farazi *et al.*, 2001). These modifications are sufficient for localization of proteins such as the TrxR1v3 thioredoxin reductase in mammals to the cytosolic side of the plasma membrane (Cebula *et al.*, 2013). Thus, fatty acid attachment to the N-terminal MAC or MGC of shortJAS and JAS-LIKE, and other JR proteins, may regulate plasma membrane localization of these proteins. This is consistent with the observations that the short version of MtrJR1 did not localize to the plasma membrane, as MtrJR1 is the only JR protein to differ in the region with the sequence MQC.

Both, the longJAS and shortJAS proteins also localize to the tonoplast (Fig. 2) and altering the N-terminal sequence of longJAS did not alter this localization (Fig. 3). This suggests that the JAS protein may also contain a tonoplast localization signal that is independent of the N-terminal region and competes with the N-terminal signals. While a number of other peripheral membrane proteins have been shown to localize to the tonoplast (Shimaoka *et al.*, 2004), little is known about the mechanisms and signals responsible for such localization, although protein–protein interactions and fatty acid attachment are again likely to play a role.

Overall, our analysis of the localization of the JAS protein revealed that the N-terminal of the long protein forms a basic AA rich Golgi localization signal. When this region is absent in the short protein, a plasma membrane localization signal is exposed. Both forms of JAS also localize to the tonoplast, suggesting that there is also a signal for tonoplast localization. Thus, the localization of JAS to multiple subcellular compartments is due to the presence of multiple localization signals.

### Leaky ribosome scanning leads to the production of the two forms of JAS

In roots cells the two forms of JAS likely arise from a single genomic region, shown by proteins encoded by the *JASg-GFP* construct localizing to both the Golgi and plasma membrane. There are multiple ways a single gene can encode multiple forms of a protein (Small *et al.*, 1998; Silva-Filho, 2003, Mackenzie 2005, Carrie and Whelan, 2013). One mechanism is leaky ribosome scanning utilizing multiple TISs. In this mechanism, the ribosome scans from the 5′-cap of the mRNA but only sporadically recognizes the first AUG, often in a suboptimal Kozak sequence, to initiate translation, while on other occasions the ribosome continues scanning until it reaches another AUG with a more optimal Kozak sequence (Kozak, 1987, 2002). We propose that the two forms of JAS are generated by leaky ribosome scanning and alternative TISs based on (i) the coding region of the *longJASc-GFP* and *JASg-GFP* constructs being identical but the proteins produced displaying different localization patterns, making proteolytic cleavage unlikely, (ii) expression of longJASg–GFP and longJASc–GFP with different promoters and 5′-UTRs resulting in similar localization patterns, making transcriptional control and alternative splicing unlikely, (iii) the suboptimal Kozak context of the first AUG in the *JAS* mRNA followed by a context closer to the optimal Kozak at the AUG at position +85, (iv) the ability of ATGs at both +1 and +85 to initiate translation in luciferase assays and in Arabidopsis seedlings as assessed by ribosome protection data (Juntawong *et al.*, 2014), and (v) the lack of plasma membrane localization of JASg–GFP when the four nucleotides upstream of the first AUG are altered (*CTTCJASg-GFP*) likely due to enhanced translation at the first AUG reducing initiation from the downstream site.

Leaky ribosome scanning and alternative TISs have been described for a range of proteins in animals, plants, and viruses. The alternative N-termini often impact upon the localization of the protein (Wamboldt *et al* 2009; Yogev and Pines, 2011; Ryabova *et al.*, 2006). For example in Arabidopsis the chloroplast and mitochondrial localization of the Lon1 protease (Daras *et al.*, 2014), the organelle DNA polymerase POLγ2 (Wamboldt *et al.*, 2009), and the mitochondrial and nuclear localization of the DNA ligase AtLIG1 (Sunderland *et al.*, 2004) result from the use of alternative TISs. While JAS is similar in that the alternative N-termini alters subcellular location, it differs from many other examples in that JAS localizes to different membranes rather than different organelles.

### Significance of the multiple forms of JAS

As the JASg–GFP protein localizes to the Golgi and plasma membrane in Arabidopsis root cells, we conclude that the long and short forms of JAS are both present in these cells. Additionally, published ribosome protection data suggest both forms are translated in seedlings, although overall expression of JAS is low (Supplemental Fig. S6; Juntawong *et al.*, 2014). The functional importance of having two forms of JAS is not known, especially as there is no clear root phenotype in *jas* mutant plants. It may be that the two forms have different molecular functions or that the same function is required in the Golgi and the plasma membrane. It does, however, appear that the choice of TISs in seedlings is regulated with translation from the AUG at position +85 producing the shortJAS favored under normal growth condition and the AUG at +1 giving longJAS favored under anoxic conditions. Regulated selection of AUGs during scanning and the discrimination between optimal and suboptimal Kozak contexts can be influenced by factors such as the abundance and activity of certain translation initiation factors (Pestova and Kolupaeva, 2002; Jackson *et al.*, 2010). For example, binding of the translation initiation factor elF4A has been proposed to influence selection of the TIS of the Arabidopsis *POLγ2* mRNA (Wamboldt *et al.*, 2009). It is therefore possible that differential activity of translation initiation factors in seedlings under stress conditions such as hypoxia influences the selection of AUG and subsequently the ratio of the long and short versions of the JAS protein. Indeed, control of translation initiation has long been recognized as a response to hypoxic conditions in plants (Branco-Price *et al.*, 2005, 2008; Muñoz and Castellano, 2012).

In male meiotic cells the JAS protein is required to maintain an organelle band across the equator of the cell during the second division to ensure correct spindle position and reduced meiotic products (Brownfield *et al.*, 2015). The shortJASc–GFP protein was predominantly localized to this organelle band, while the longJASc–GFP protein was in other areas of the cell as well as the organelle band. In previous work we found that JASg–GFP localized almost exclusively to the organelle band (Brownfield *et al.*, 2015). The similarity in the localization of the shortJASc–GFP and JASg–GFP in meiocytes strongly suggests that the shortJAS protein is the predominant form in meiotic cells. Consistently, the lack of JASg–GFP outside the organelle band reveals that little, if any, of the longJAS protein is produced during meiosis. The shortJAS in meiocytes is likely to be present in plasma membrane-containing vesicles in the organelle band, as shortJAS localizes to the plasma membrane in roots and the plasma membrane marker is present in the organelle band of meiotic cells, overlapping with JASg–GFP (Brownfield *et al.*, 2015). In agreement with shortJAS being the predominant form during meiosis, our complementation analysis showed it is also the functional form required for maintenance of the organelle band and the production of haploid meiotic products.

The predominance and functional importance of the short JAS protein in meiocytes indicates that the choice of TIS is regulated in these cells to favor initiation from the ATG at +85. A role for translational regulation in animal (Bettegowda and Wilkinson 2010) and yeast (Berchowitz *et al* 2013) meiosis is well documented, but little is known about translational regulation of plant meiosis. This is, a far as we are aware, the first report of the use of alternative TISs being regulated in plant meiosis. As this regulation is likely to rely on differences in the activity of elongation factors, it is expected that the choice of TIS in other transcripts will be similarly regulated in meiotic cells in plants. Indeed, ribosome profiling has shown the use of alternative TISs is common in yeast meiosis (Brar *et al.*, 2012).

The observation that production of the short form of JAS during meiosis is regulated suggests a possible link between JAS regulation and the occurrence of unreduced pollen during stress conditions. A link between stress and the formation of unreduced male gametes through spindle misplacement, similar to the *jas* mutant, has long been observed (reviewed in De Storme and Geelen, 2014). Additionally, stress can influence the activity of translation elongation factors and the choice of TIS (Pestova and Kolupaeva, 2002; Jackson *et al.*, 2010). Thus, it is possible that stress during meiosis alters elongation factor activity such that the early AUG in the *JAS* mRNA is preferentially selected and the long protein translated, as was observed in seedlings under hypoxic stress. This alteration in TIS would lead to a reduction in the amount of the short protein that is functional during meiosis, causing a disturbance in the organelle band and spindle misplacement, resulting in unreduced pollen.

## Supplementary data

Supplementary data are available at *JXB* online.

Fig. S1. Predicted secondary structure of the JAS protein.

Fig. S2. Subcellular localization of stopJASg–GFP.

Fig. S3. Subcellular localization of cDNA-encoded JAS–GFP.

Fig. S4. Plasmolysed Arabidopsis root cell with JAS-LIKE–GFP.

Fig. S5. Subcellular localization of GFP with the N-terminal 28 AA of longJAS.

Fig. S6. Ribosome protection data for *JAS*.

Table S1. Primers used in this study.

Table S2. Gene identifiers for JR proteins shown in Fig. 4.

## Supplementary Material

Supplementary Tables
