# Supplementary material for: The meiotic regulator JASON utilizes alternative translation initiation sites to produce differentially localized forms
Source: J Exp Bot. 2017 Jul 11;68(15):4205–17. doi: 10.1093/jxb/erx222 (PMC5853252; doi:10.1093/jxb/erx222)
Supplement: Supplementary Tables [file erx222_suppl_supplementary_tables.pdf]

Table S1. Primers used to in this study. Gateway *att* site are in italics and restriction enzyme sites are in bold.

**Name**	**Sequence 5' to 3'**	**Role**
JAS F	TCTTCCCATTTTCACTCATGG	Screening for T-DNA insertion
JAS R	TCTGTACGAAAGCCAAGGAGA	Screening for T-DNA insertion
SAIL T-DNA LB2	GCTTCCTATTATATCTTCCCAAATTACCAATACA	Screening for T-DNA insertion in JAS with JAS screening F
JASg F	GCCTTTCTTCCCCACTCCTACAG	JASg
JASg R	GAAGGAAACTACCGAGTTTGGTTGC	JASg
shortJASg F	GGAAATCAAATAATACGAGACGACTGTTGACGAACCGAAACC	Site directed mutagenesis for shortJASg
shortJASg R	GGTTTCGGTTCGTCAACAGTCGTCTCGTATTATTTGATTTCC	Site directed mutagenesis for shortJASg
longJASg F	CAGTCTGTAGAACCCTTGCTTGTTTTCTCG	Site directed mutagenesis for longJASg
longJASg R	CGAGAAAACAAGCAAGGGTTCTACAGACTG	Site directed mutagenesis for longJASg
stopJASg F	CGATTTATCAGTCTAGAGAGCCATGGC	Site directed mutagenesis for stopJASg
stopJASg R	GCCATGGCTCTCTAGACTGATAAATCG	Site directed mutagenesis for stopJASg
mutlongJASg F	CAAATGATATGGACGACCTGTTGATGAACGACAACCTAAGC	Site directed mutagenesis for mutlongJASg
mutlongJASg R	GCTTAGGTTGTCGTTCATCAACAGGTCGTCCATATCATTTG	Site directed mutagenesis for mutlongJASg
CTTCJASg F	TTCATCAACAGTCGTCTCATGAAGTTTGATTTCCTCAAGAGCGAAACAGTGATTGACC	Site directed mutagenesis for CTTCJASg
CTTCJASg R	GGTCAATCACTGTTTCGCTCTTGAGGAAATCAAACTTCATGAGACGACTGTTGATGAA	Site directed mutagenesis for CTTCJASg
longJASc F	*GGGGACAAGTTTGTACAAAAAAGCAGGCTTC*ATGAGACGACTGTTGATGAAC	longJASc, JAS NT, JASNT-JAS-LIKE
Short JASc F	*AAAAAAGCAGGCTTC*ATGGCTTGTTTTCTCGACTGC	shortJASc
JASc R	*CAAGAAAGCTGGGTT*GAAGGAAACTACCGAGTTTG	longJASc, shortJASc
JAS-LIKE F	*AAAAAAGCAGGCTTC*ATGGGTTGTCTCTTCGGTTGC	JAS-LIKEc
JAS-LIKE R	*CAAGAAAGCTGGGTT*AAAGGAAATGACTGAATGTCG	JAS-LIKEc, NT-JAS-LIKE
NTJAS-LIKE F	GACAACCCATGGCTCTACAGACTGATAAATCG	First PCR for NT-JAS-LIKE
NTJAS-LIKE R	CTGTAGAGCCATGGGTTGTCTCTTCGGTTGC	First PCR for NT-JAS-LIKE
NTJAS-F	*GGGGACAAGTTTGTACAAAAAAGCAGGCTTC*ATGAGACGACTGTTGATGAAC	JAS NT (for NT-GFP), NT-JAS-LIKE
NTJAS-R	*GGGGACCACTTTGTACAAGAAAGCTGGGT*AGTCGAGAAAACAAGCCATGGC	JAS NT (for NT-GFP)
promUBQ14 F	*GGGGACAACTTTGTATAGAAAAGTTG*ATCCGAACAGAGTTAAACCGG	UBQ14 promoter
promUBQ14 R	*GGGGACTGCTTTTTTGTACAAACTTG*AAACTGAGATTAATCGCTTGG	UBQ14 promoter
Long MtrJR1 cDNA F	*GGGGACAAGTTTGTACAAAAAAGCAGGCTTC*ATGTTGTGGCTTGTGAAACGTGGA	longMtrJR1c
Short MtrJR1 cDNA F	*GGGGACAAGTTTGTACAAAAAAGCAGGCTTC*ATGCAGTGCTTCTTCGGAATCAGAG	shortMtrJR1c
MtrJR1 cDNA R	*GGGGACCACTTTGTACAAGAAAGCTGGGTG*CTCCTCAATCTCCTCAAATGCCA	longMtrJR1c, shortMtrJR1c
JAS LUC F	**GGCAAGCTT**TCACTGTTTCGCTCTTGAGG	5'end JAS for luciferase assay with HindIII site
JAS LUC R	**GGCGGATCC**ATTGGCGAGTGAAGAATGAGA	5'end JAS for luciferase assay with BamHI site

Table S2. Gene identifiers for JR proteins used in Fig. 4.

**Abbreviation**	**Species**	**Gene Identifier**
AthJAS	*Arabidopsis thaliana*	At1G06660
AthJL	*Arabidopsis thaliana*	At2G30820
AlyJR1	*Arabidopsis lyrata*	481999
AlyJR2	*Arabidopsis lyrata*	887868
CruJR1	*Capsella rubella*	Carubv10012445m
CruJR2	*Capsella rubella*	Carubv10023398m
BraJR1	*Brassica rapa*	Bra015516
BraJR2	*Brassica rapa*	Bra022814
CpaJR1	Carica papaya	evm.model.supercontig_50.118
TcaJR1	*Theobroma cacao*	Thecc1EG001947t1
CclJR1	*Citrus clementine*	Ciclev10031282m
PtrJR1	*Populus trichocarpa*	Potri.002G057000
PtrJR2	*Populus trichocarpa*	Potri.005G205700
MesJR1	*Manihot esculenta*	cassava4.1006366
MesJR2	*Manihot esculenta*	cassava4.1007124m
MtrJR1	*Medicago truncatula*	Medtr5g090900
MtrJR2	*Medicago truncatula*	Medtr3g450760
GmaJR1	*Glycine max*	Glyma14g03450
GmaJR2	*Glycine max*	Glyma02g45320
GmaJR3	*Glycine max*	Glyma08g41940
GmaJR4	*Glycine max*	Glyma18g13680
CsaJR1	*Cucumis sativus*	Cucsa.179850
PpeJR1	*Prunus persica*	ppa024302m
VviJR1	*Vitis vinifera*	GSVIVT01020830001
SlyJR1	*Solanum lycopersicum*	Solyc07g045010.2.1
SlyJR2	*Solanum lycopersicum*	Solyc12g006270
AcoJR1	*Aquilegia coerulea*	Aquca12500018
AcoJR2	*Aquilegia coerulea*	Aquca02600365

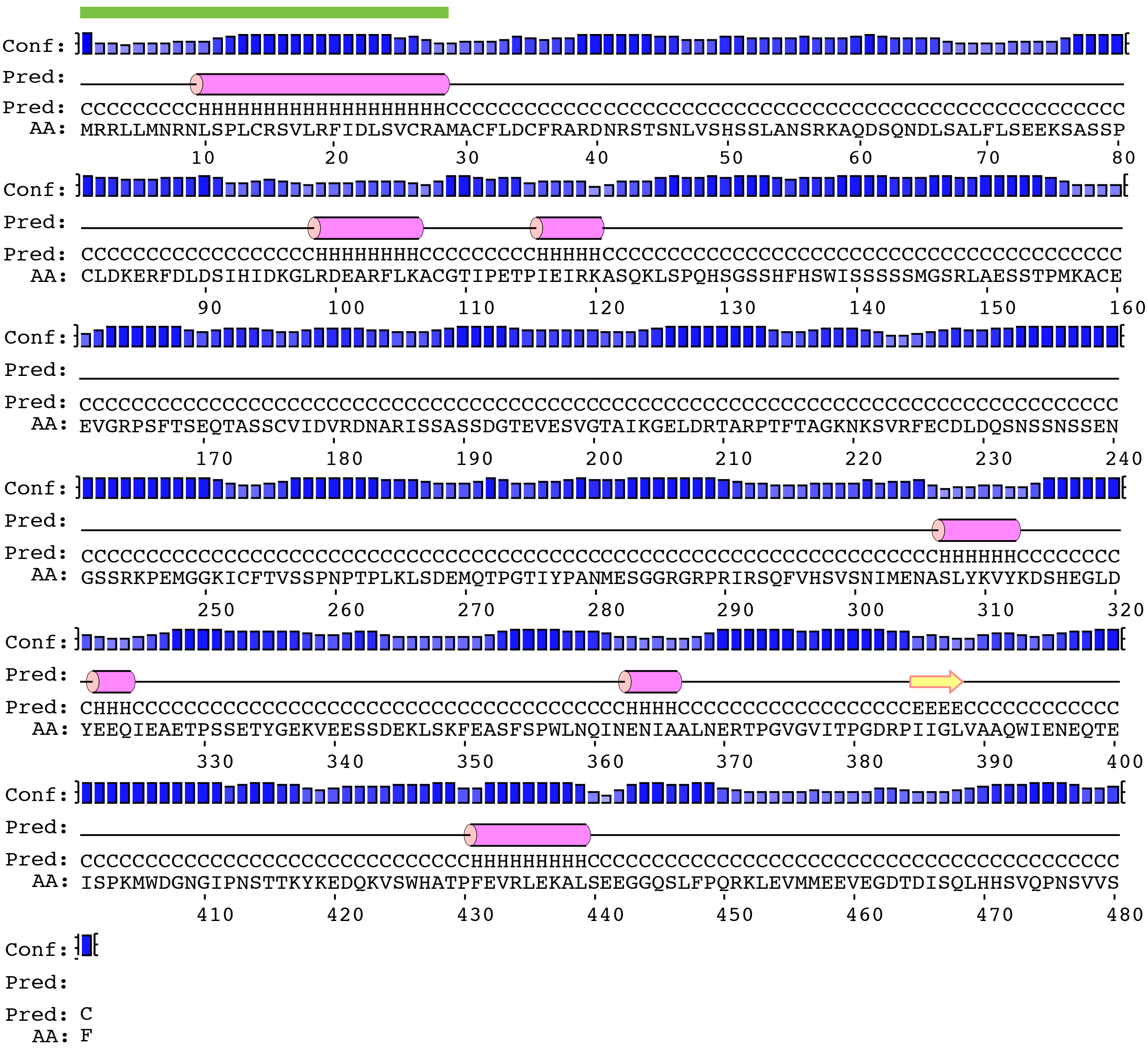

Fig. S1. Predicted secondary structure of the JAS protein. The AA sequence of JAS was analysed for localization signals and secondary structure. An N-terminal mitochondrial transit peptide (green rectangle) was predicted by TargetP and SLP-Local. The secondary structure of JAS was predicted by PSIPRED to be largely unstructured (black line) with some α-helices (pink barrel) and one β-strand (yellow arrow). Conf indicates the confidence of the prediction, Pred is the predicted secondary structure and AA the shows the target sequence.

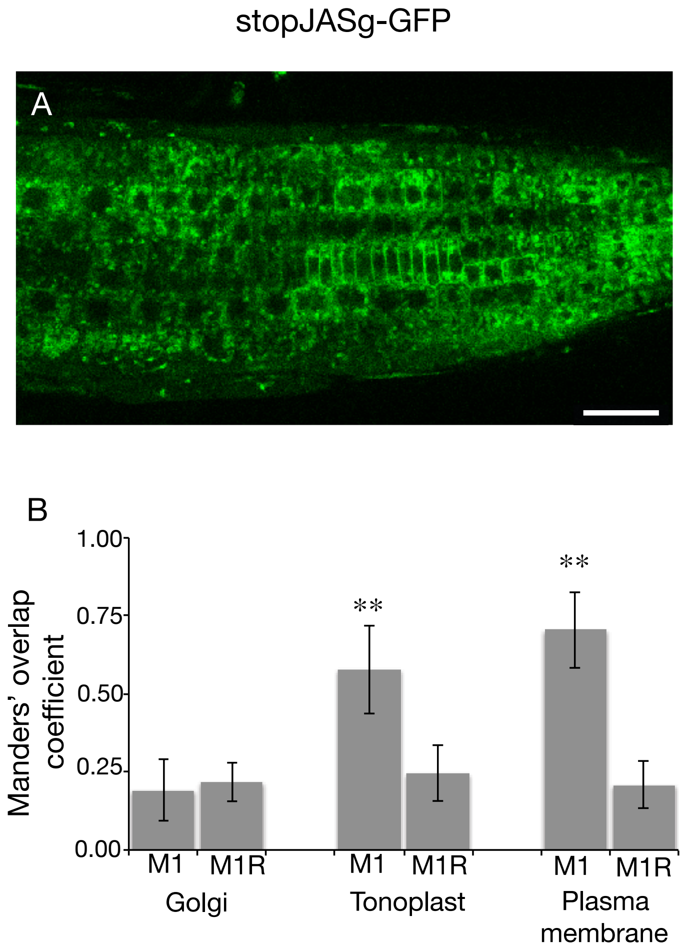

Fig. S2. Subcellular localization of stopJASg-GFP. CLSM was used to view the subcellular location of JAS without the N-terminal peptide (stopJASg-GFP) in the meristematic region of Arabidopsis roots. (A) GFP fluorescence in whole root tips for stopJASg-GFP. (B) Quantification of co-localization for stopJASg-GFP and markers for the Golgi, tonoplast and plasma membrane. For each combination the mean (± standard deviation) Manders' overlap coefficient 1 value (M1) from 20 images is shown along with the average (± standard deviation) for the same red image with the green image randomized (M1R). ** indicates a that the M1 value is significantly higher than the M1R value (*t*-test, P<0.001). Scale bar = 20 µm.

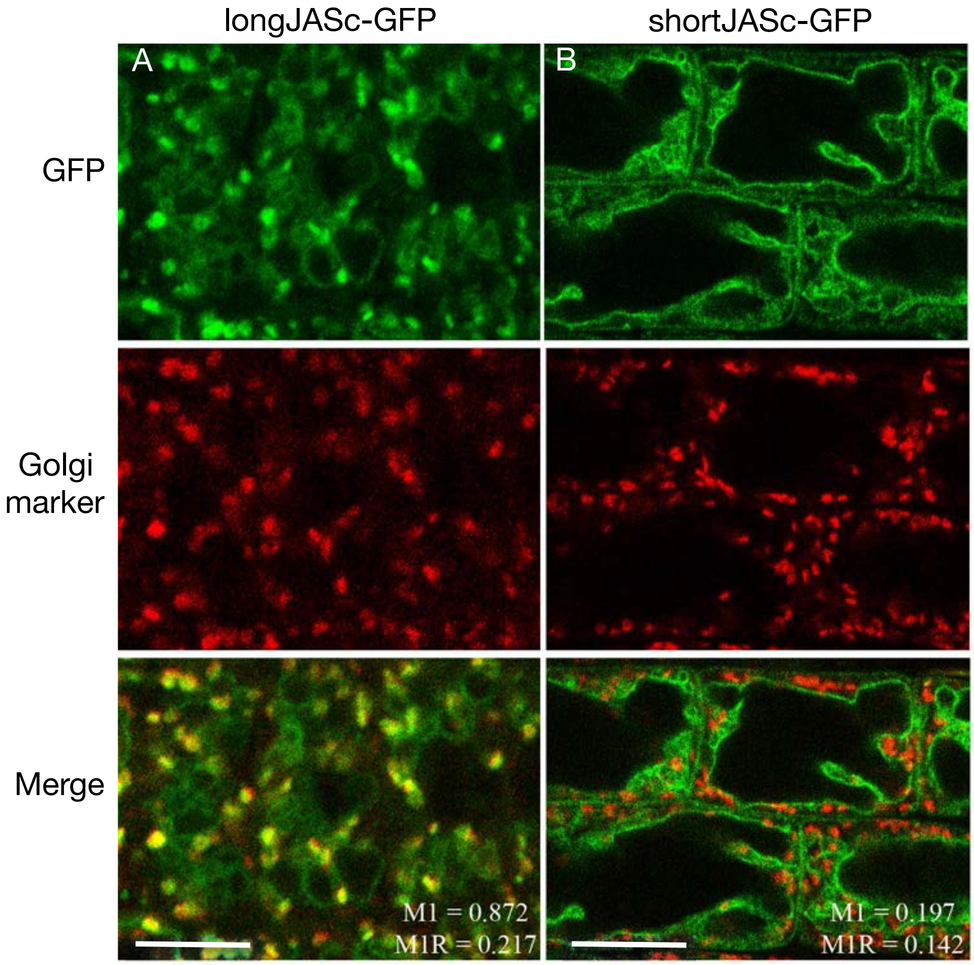

Fig. S3. Subcellular localization of cDNA encoded JAS-GFP. (A,B) CLSM images of meristematic root cells of Arabidopsis showing JAS-GFP (A) from longJASc-GFP (starting at the first ATG) or (B) shortJASc-GFP (starting at the second ATG). The top panel shows GFP fluorescence (green), the middle panel shows Golgi marker fluorescence (red) and in the bottom panel co-localization can be visually assessed by a yellow color in merged images. The Manders' overlap coefficient 1 for each pair of images (M1) and when the green image is randomized (M1R) is indicated on the merged image. Scale bar = 5 µm.

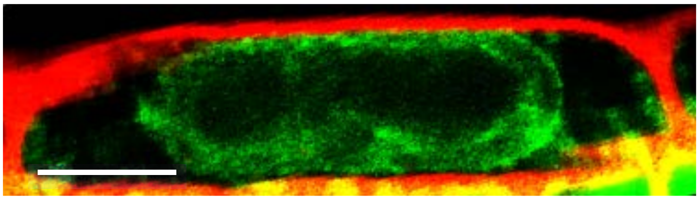

Fig. S4. Plasmolyzed Arabidopsis root cell with JAS-LIKE-GFP. CLSM image of an Arabidopsis root cell expressing JAS-LIKE-GFP (green) after incubation for 1h in mannitol. The plant cell wall is stained with red with propidium iodine. Scale bar = 5 µm.

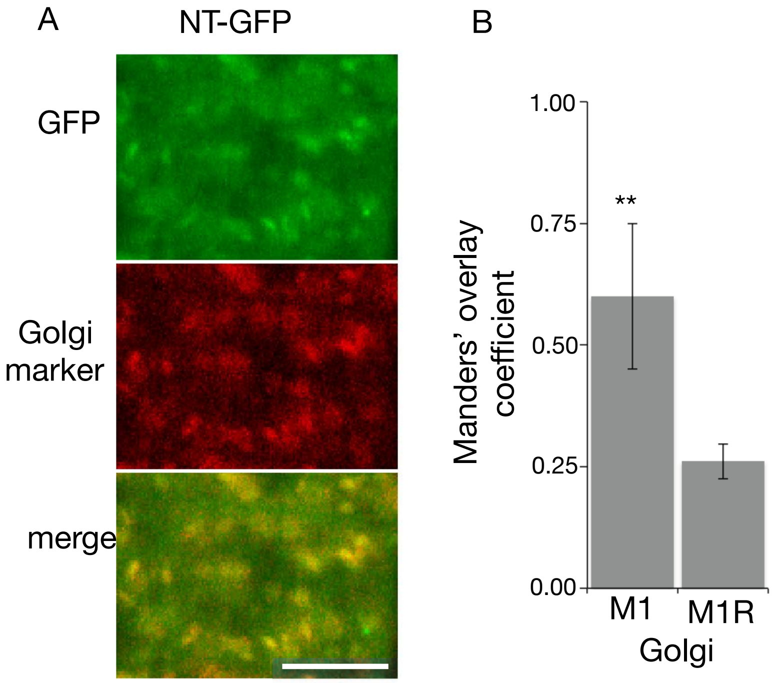

Fig. S5. Subcellular localization of GFP with the N-terminal 28 AA of longJAS. The N-terminal region of longJAS was cloned upstream of GFP (NT-GFP) and localization analysed in Arabidopsis root cells. (A) CLSM images of Arabidopsis meristematic roots cells with NT-GFP. Top panel shows GFP fluorescence (green), the middle panel shows Golgi marker fluorescence (red) and in the bottom panel co-localization can be visually assessed by a yellow color in merged images. (B) Quantification of co-localization of NT-GFP and the Golgi marker. For each combination of GFP and marker the average (± standard deviation) Manders' overlap coefficient 1 value (M1) from 20 images is shown (M1) along with the average (± standard deviation) for the same red image with the green image randomized (M1R). ** indicates that the M1 value is significantly higher than the M1R value (*t*-test, $P<0.001$). Scale bar = 5 μm.

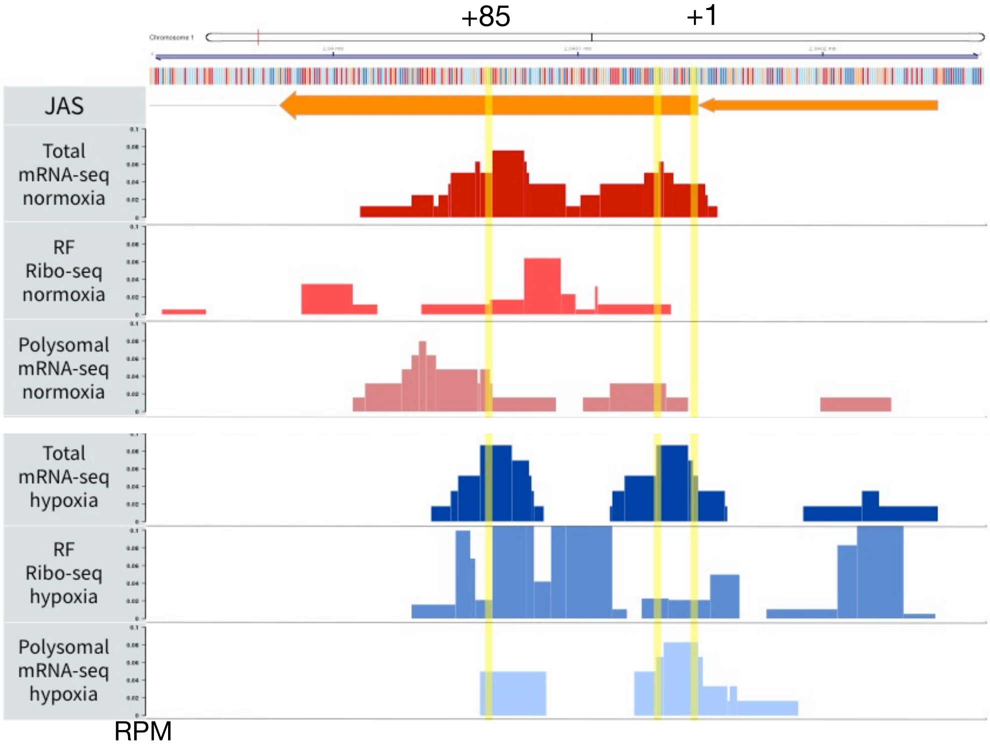

Fig. S6. Ribosome protection data for *JAS*. Total RNA read coverage, polysomal mRNA and ribosome footprint (RF) at the 5'UTR and first exon of *JAS*, under normal oxygen (normoxia) amd oxygen deprived (hypoxia) conditions. Data from Juntawong et al., 2014. Note JAS is in the reverse orientation. The three potential start codon positions are highlighted in yellow through all samples with those at +1 and +85 indicated above. The 5' UTR of *JAS* is indicated by a narrow orange arrow and the exon by a thick orange arrow. The read coverage was normalized by reads per million (RPM).
